# Comparing isolated soy protein with flaxseed oil vs isolated soy protein with corn oil and wheat flour with corn oil consumption on muscle catabolism, liver function, blood lipid, and sugar in burn patients: a randomized clinical trial

**DOI:** 10.1186/s13063-018-2693-5

**Published:** 2018-06-04

**Authors:** Siavash Babajafari, Abdollah Hojhabrimanesh, Zahra Sohrabi, Mehdi Ayaz, Ali Noorafshan, Atefeh Akrami

**Affiliations:** 1 Nutrition Research Center, School of Nutrition and Food Sciences, Shiraz University of Medical Sciences, Shiraz, Iran; 2 Burn Research Center, Shiraz University of Medical Sciences, Shiraz, Iran; 3 Histomorphometry and Stereology Research Center, Shiraz University of Medical Sciences, Shiraz, Iran

**Keywords:** Isolated soy protein, Flaxseed oil, Muscle catabolism, Weight gain, Randomized controlled trial

## Abstract

**Background:**

There is controversy regarding whether increasing isolated soy protein (ISP) with or without flaxseed oil (FO), as functional foods, would lead to reduce muscle catabolism and cachexia in burn patients.

**Methods:**

One hundred and eighty-eight patients were assessed for eligibility in this randomized controlled trial. Of these, seventy-three eligible patients (total burn surface area 20–50%) were randomly assigned to three groups, labeled as Control (wheat flour [WF] + corn oil [CO]), ISP + FO, and ISP + CO, to receive these nutrients for three weeks. Weight, body mass index (BMI), serum hepatic enzymes (alanine transaminase [ALT], aspartate transaminase [AST], alkaline phosphatase [ALP]), systemic inflammatory response syndrome (SIRS), 24-h urinary urea nitrogen excretion (UUN), serum creatinine, 24-h urinary creatinine (UUC) excretion, fasting blood sugar (FBS), triglyceride (TG), and cholesterol were measured.

**Results:**

Using analysis of covariance models in the intention-to-treat population (*n* = 73), we found that at three weeks, patients in the ISP groups had lost significantly less in weight and BMI compared to those in the control group (all *P* < 0.01). Nitrogen retention and serum creatinine (primary outcomes) increased significantly in the ISP groups compared with the control group. Even after controlling for potential covariates in ANCOVA models, changes in these indices were still statistically significant (*P* = 0.008 and *P* = 0.005 for nitrogen balance and serum creatinine, respectively). However, no such significant differences were found between the ISP groups. On the other hand, 24-h UUN, and UUC excretion, serum hepatic enzymes, FBS, TG, and cholesterol were not significant between the groups (*P* > 0.05).

**Conclusion:**

ISP and FO compared to WF and CO reduced muscle catabolism and increased body weight in burn patients.

**Trial registration:**

Iranian Registry of Clinical Trials, IRCT2014051817740N1. Registered on 27 June 2014.

## Background

Burn injuries lead to whole-body catabolism, significantly elevated resting energy expenditure (REE), and multi-organ dysfunction. Burn patients have liver dysfunction, severe muscle catabolism, increased protein degradation, and insulin resistance [1, 2]. Burns of > 20% of the total body surface area result in malnutrition, poor wound healing, muscle wasting, severe cachexia, frequent infections, and anemia [3–5]. Nutritional support is recognized as one of the most significant aspects of care for burned patients [6]. Isolated soy protein (ISP) contains high quantities of amino acids, such as glutamine, arginine, and branched-chain amino acids (BCAA) [7]. The beneficial effects of these amino acids have been demonstrated on other studies with critical conditions and wound healing in the other diseases [8–14]. Moreover, flaxseed oil (FO) is a rich source of omega-3 fatty acids with anti-inflammatory effects on burn-induced inflammation [15]. In general, omega-3 fatty acids have been found to be beneficial in wound healing [16] and improving immune function [17, 18]. Other studies with different clinical conditions have shown that ISP improves nitrogen balance, muscle protein synthesis, serum albumin, hepatic enzymes, blood sugar, triglyceride, and cholesterol in the other diseases [19–22]. However, despite the escalating literature supporting the potential effect of FO and ISP as wound-healing agents in experimental animals, so far there have been no randomized controlled trials (RCTs) in humans assessing the effects of ISP and FO on burn patients. To shed light on these controversies, we decided to design a RCT study to evaluate these functional foods on muscle catabolism, liver function, blood lipid, and sugar in burn patients.

## Methods

### Patients

As part of our former study [23], we collected information of burn patients from the Institute of Burn Research of Qutb al-Din hospital, affiliated with Shiraz University of Medical Sciences. Eligible patients were required to have the following criteria: aged 15–60 years; a body mass index (BMI) of 18.0–30 kg/m^2^ for individuals aged > 18 years and – 2 SD to + 2 SD percentile for those aged < 18 years [24]; total burn surface area (TBSA) of 20–50%; and the ability to understand the study protocol and provide written informed consent. Exclusion criteria included: renal or hepatic failure; severe inhalation injury; excessive hemorrhaging; allergy to soy; use of omega-3 fatty acids in the previous month; in need of parenteral or enteral nutritional support before randomization; and > 3 kg weight change over the preceding three months. The ethics board of Shiraz University of Medical Sciences, Shiraz, Iran approved the study protocol (reference no. CT-92-6878). All procedures were followed in accordance with the ethical standards laid down in the Declaration of Helsinki and its later amendments [25]. All patients or their close relatives provided written, informed consent before enrollment and we took consent to participate in the study from their parent or legal guardian in the case of patients aged < 16 years. This trial was registered at http://en.irct.ir/trial/16230 as IRCT2014051817740N1.

### Study design

In this double-blind RCT, allocation of participants to treatment and control groups was performed by permuted block randomization. Each eligible patient received a randomization number which was determined by a computer-generated schedule. The investigator and patients were blinded to the treatment condition, then a randomization table was generated by the method of random permuted blocks. People who were operationally independent from the study investigator performed the study randomization. Patients’ data collected during this trial was kept confidential and locked in a secure area. Randomization codes of the study were opened only after all participants complete the study protocol. To maintain and guarantee blinding, treatment products and control products were identical in appearance. Using simple randomization [26], participants were assigned in a 1:1:1 fashion to three equal groups, labeled as ISP + FO (Group A), ISP + corn oil (CO) (Group B), and wheat flour (WF) + CO (Control group) for three weeks. Because of the large perturbation in water compartments of burn body patients that occurred after admission time, fasting venous blood samples were taken on the morning of day 4 and day 25. During the study, from the 188 patients who were initially screened, 73 met the inclusion criteria and entered the intervention. The 25 patients in Group A received snacks with FO and ISP daily for three weeks. The 24 patients in Group B received snacks with CO and ISP daily for three weeks. The 24 patients in Group C, the control group, received snacks with CO and WF every day for three weeks. Participants were asked to avoid nutritional supplements, isoflavones, or phytate-rich foods during the treatment period. All patients received conventional wound management and fluid replacement. The intervention lasted for 21 days, from day 4 after admission until day 25. The study was performed from June 2014 through December 2014.

### Snacks

Snacks were provided in the form of cookies. Cookies were prepared with 50 g ISP powder (SUPRO® Isolated Soy Protein, DANISCO, Denmark) and 30 g FO (Abkar Golestan Cultivate & Industry Co, Iran) (Group A), 50 g ISP powder and 30 g CO (Group B), and 50 g WF and 30 g CO (Group C, control product). In the control group, CO and WF were selected as substitutes for FO and ISP, respectively. The doses of ISP [27] and FO were determined according to previous studies [28]. Other materials including milk, yogurt, rice flour, sugar, and water were used in similar quantities in cookies of the three groups and were also similar in protein and energy (Table 1). To improve the appearance and acceptance of the cookies, three different types of decoration were used. These decorations contained honey, concentrated date syrup, and chocolate. To ensure blinding, the cookies given to all three groups were of similar appearance each day. The amounts of energy, fat, and carbohydrates were almost similar in the cookies of the three groups. Since this study was a double-blind RCT, cookies were packed in similar packaging for all three groups. All patients received the conventional nutritional support of the Qutb al-Din hospital. This included 40 kcal/kg/day energy average (35–45 kcal/kg/day based on burn extent, number of surgeries, and body weight) and 1.5 g/kg/day protein average (1.2–1.5 g/kg/day) according to previous studies [29, 30]. There was no difference in the average daily calorie and nitrogen intake between individuals of the three groups. The diet of the Qutb al-Din hospital contained on average 24% protein, 27% fat, and 49% carbohydrate for all patients.

**Table 1:** Ingredients of cookies with three decorations (honey, concentrated date syrup, and chocolate)

Ingredients (g/230 g cookie/day)	Group A ISP + FO (*n* = 25)	Group B ISP + CO (*n* = 24)	Group C Control (*n* = 24)
ISP	50	50	0
FO	30	0	0
WF	0	0	50
CO	0	30	30
Sugar	35	35	35
Medium-fat yogurt	27	27	27
Medium-fat milk	50	50	50
Rice flour	20	20	20
Honey	20	20	20
Concentrated date syrup	19	19	19
Chocolate	20	20	20
Pistachios	10	10	10
Protein:			
Honey	47.83	47.76	10.49
Chocolate	48.89	48.46	11.54
Concentrated date syrup	48.34	48.1	10.99
Kcal			
Honey	788.4	783.9	762.7
Chocolate	803.1	798.6	777.4
Concentrated date syrup	785.8	781.3	760.1

*ISP* isolated soy protein, *FO* flaxseed oil, *WF* wheat flour, *CO* corn oil

### Anthropometric measurements

Knee height is correlated with stature and, until recently, was the preferred method for estimating height in bedridden patients Knee height was measured as a surrogate measure of height. Knee height was measured in the recumbent position from the top of the patella to the bottom of the heel pad with the knee flexed at 90°. Measurements were recorded to the nearest 0.1 cm. A nomogram was used to convert the knee height to the height with a precision of ± 6 cm (90% confidence limits) [31, 32]. Weight was measured using an electronic scale (Beurer PS160, Germany) to the nearest 0.1 kg. All anthropometric measurements were performed twice and the average of two measurements was considered. If the difference between two measurements was > 1.0 cm or 0.1 kg, a third measurement was taken and the two closest values were averaged.

### Outcome measurements and systemic inflammatory response syndrome (SIRS)

We assessed the muscle catabolism of patients by nitrogen balance and 24-h urine creatinine as primary outcomes, with BMI, weight changes, and liver function indices as secondary outcomes. Fasting venous blood samples were obtained at the start and end of the study phase. The participants were asked to fast overnight for 12–14 h. Twenty-four–hour urine samples were collected on the fourth day of admission and at the end of the study. Serum was frozen at − 20 °C until the final analysis. Cell blood count (CBC) was measured by using a cell counter Sysmex KX21 machine. The bromocresol green method (Pars Azmoon Co., Tehran, Iran) was used for measuring serum hepatic enzymes (including ALT, AST, and ALP). Serum and urine concentrations of creatinine were measured by the colorimetric methods (Pars Azmoon Co., Tehran, Iran). FBS, TG, and cholesterol were measured by enzymatic methods (Pars Azmoon Co., Tehran, Iran). We calculated the total lymphocyte count (TLC) per cubic millimeter by multiplying WBC by the absolute percentage of total lymphocytes. Nitrogen balance was calculated each day as follows: nitrogen balance (gN/kg) = nitrogen intake (gN/kg) – total nitrogen excretion (gN/kg). The nitrogen intake (gr/N) was estimated as protein intake (gr) divided by 6.25 based on the calculated 24-h dietary recall from each patient [33, 34].We collected 24-h urine by using a plastic bag connected to a urinary catheter for three days. On each day, duplicate samples (each approximately 10 mL) were collected from pooled urine after stirring adequately and storing at − 20 °C for later use. The mean value of duplicates was used for further analysis to reduce measurement errors. The concentration of urinary urea nitrogen was measured using the urease method [35]. Criteria for SIRS were established in 1992 as a part of the American College of Chest Physicians/Society of Critical Care Medicine Consensus Conference. The conference concluded that the manifestations of SIRS include, but are not limited to, the following: body temperature < 36 °C or > 38 °C, heart rate > 90 beats per minute, tachypnea (high respiratory rate) with > 20 breaths per minute, an arterial partial pressure of carbon dioxide < 32 mmHg, white blood cell count < 4000 cells per cubic millimeter or > 12,000 cells per cubic millimeter, or the presence of > 10% immature neutrophils (band forms). SIRS is diagnosed when two or more of these criteria are present [36].

### Statistical analysis

SPSS statistical software package version 16 (SPSS, Inc.) was used for statistical analyses. Paired t-test was used for analyzing changes in each group during the treatment phase for normally distributed data; for skewed data, we used Wilcoxon signed-rank test. For comparing the changes in study variables between groups, an analysis of covariance model with treatment as the main effect and baseline parameters as covariates was performed. Bonferroni post hoc test was used to examine pairwise differences between groups to adequately adjust for multiple comparisons. *P* < 0.05 was considered statistically significant. We selected a sample size of 24 per group with assumption of nitrogen balance reduction as much as 2.5 ± 1.5 g/day by using a statistical power of 90%, a two-sided α level of 0.05, and 20% attrition rate.

McNemar’s test was used to analyze the SIRS binary variable before and after the intervention. Dietary intakes were obtained on the first day to assess the baseline nutritional status of the patients. Dietary intakes were analyzed using NUTRITIONIST 4 software, version 3.5.2 (First Data Bank, San Bruno, CA, USA).

## Results

### Participant characteristics

**Fig. 1:**
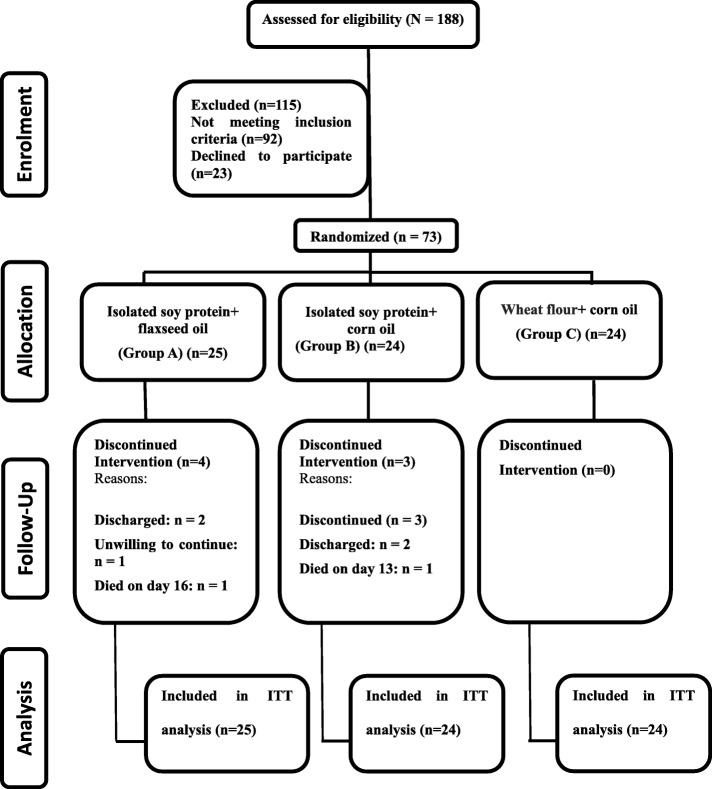
Participant *flow diagram* throughout the study. ITT intention-to-treat

Figure 1 illustrates the patient flow throughout the study. A total of 188 individuals were assessed for eligibility. Of these, 92 did not meet inclusion criteria and 23 declined to participate. Of these, nine (9.6%) discontinued intervention before the end of the intervention period (Group A: *n* = 4, Group B: *n* = 3, Control: *n* = 0). The reasons for the drop-outs were as follow: four patients were discharged from the hospital during the course of the study; two died on day 13 and day 16 due to septicemia and pulmonary embolism, respectively; and one lost the willingness to cooperate after day 5 (Fig. 1). There was no significant difference in drop-out rate among the three groups. Causes of burn included: 29 cases of kerosene fire; 24 cases of gas explosion; 11 cases of flame burns; five cases of electrical currents; and four cases from hot liquid. Sixty-six patients (50 men, 16 women) completed the study. The mean age of the patients was 32.5 ± 10.6 years. The average percentage of burn was 33.0 ± 9.7%. Table 2 shows the baseline demographic and laboratory parameters of the study participants. There were no significant differences in baseline characteristics between the groups. Moreover, no significant differences were observed in terms of baseline characteristics between study completers and those who discontinued intervention prematurely.

**Table 2:** Baseline biochemical and dietary characteristics of the study participants^a^

Variables	Group A ISP + FO (*n* = 25)	Group B ISP + CO (*n* = 24)	Group C Control (*n* = 24)	*P* value^b^
TBSA (%)	30.9 ± 1.93	32.5 ± 2.15	35.4 ± 2.09	0.28
Sex (M:F)	18:7	19:5	17:7	0.73
Age (year)	36.8 ± 2.56	31.6 ± 2.31	29.3 ± 1.78	0.09
Education (year)	7.1 ± 0.87	8.0 ± 0.82	9.5 ± 0.71	0.12
Grafted patients (n)	12	13	13	0.87
SIRS patients (n)	11	11	10	0.70
SBP (mmHg)	112.9 ± 3.26	116.8 ± 3.12	113.4 ± 3.08	0.64
DBP (mmHg)	72.1 ± 2.40	73.2 ± 2.12	69.3 ± 1.69	0.36
Eating problems score^c^	1.1 ± 1.0	0.90 ± 0.8	1.4 ± 1.2	0.34
Energy intake (kcal/day)	1659.4 ± 79.35	1637.3 ± 112.28	1600.9 ± 117.88	0.92
Protein intake (g/day)	107.9 ± 6.43	103.8 ± 7.05	106.1 ± 8.16	0.92
Fat (g/day)	64.4 ± 15.5	64.9 ± 17.2	61.7 ± 22.0	0.83
SFA (g/day)	24.9 ± 6.2	23.9 ± 6.7	23.5 ± 7.4	0.77
MUFA (g/day)	22.6 ± 5.6	23.3 ± 6.4	21.8 ± 6.4	0.79
PUFA (g/day)	10.5 ± 3.2	11.5 ± 4.6	10.2 ± 5.7	0.63
Linolenic acid (g/day)	0.32 ± 0.07	0.32 ± 0.10	0.30 ± 0.09	0.67

*TBSA* total body surface area, *BMI* body mass index, *SIRS* systemic inflammatory response syndrome, *SBP* systolic blood pressure, *DBP* diastolic blood pressure, *SFA* saturated fatty acids, *MUFA* monounsaturated fatty acids, *PUFA* polyunsaturated fatty acids

^a^Data are presented as means ± SD or frequency

^b^ Between-group differences were tested with one-way ANOVA

^c^ Eating problems included difficulty in chewing, dysphagia, nausea, and vomiting. Each difficulty was scored as 1 in case of presence and 0 in case of absence, and the sum of the scores was calculated

### Effect of treatment on muscle catabolism, liver function, TG, cholesterol, and FBS indices

Any changes in markers of muscle catabolism during the treatment phase are demonstrated in Table 3.

**Table 3:** The effect of treatment on markers of muscle catabolism of burn patients during the intervention^a^

Variables	Group A ISP + FO (*n* = 25)	Group B ISP + CO (*n* = 24)	Group C Control (*n* = 24)	*P* value^b^
Primary outcomes:				
Nitrogen balance (g/day)	2.19 ± 0.73^c^ (1.83 to 2.5)	2.4 ± 0.7^c^ (2.04 to 2.72)	2.3 ± 0.9^d^ (1.89 to 2.7)	0.008
Serum creatinine (mg/dl)	+ 0.75 ± 0.24^c^ (0.55 to 0.96)	+ 0.64 ± 0.24^c^ (0.49 to 0.80)	+ 0.22 ± 0.09^d^ (0.26 to 0.70)	0.005
Secondary outcomes:				
24 h UUN (g/day)	− 1.55 ± 5.8^c^ (− 4.21 to 1.10)	− 1.10 ± 6.51^c^ (− 4.07 to 1.86)	+ 2.06 ± 5.25^d^ (− 0.15 to 4.28)	0.08
24 h Urine creatinine (g/day)	+ 0.75 ± 0.24^c^ (0.55 to 0.96)	+ 0.64 ± 0.24^c^ (0.50 to 0.80)	+ 0.5 ± 0.2^d^ (0.26 to 0.73)	0.16
Weight (Kg)	− 1.71 ± 1.3^c^ (0.55 to 0.96)	−3.01 ± 2.17^c^ (0.55 to 0.96)	−6.00 ± 2.7^d^ (0.55 to 0.96)	< 0.0001
BMI (Kg/m^2^)	− 0.55 ± 0.44^c^ (0.55 to 0.96)	− 1.16 ± 1.00^c^ (0.55 to 0.96)	− 2.14 ± 0.94^d^ (0.55 to 0.96)	< 0.0001
TLC (%)	+ 0.75 ± 0.24^c^ (− 0.72 to 0.83)	+ 0.64 ± 0.24^c^ (− 1.36 to 0.72)	+ 0.5 ± 0.2^d^ (− 1.81 to − 0.11)	0.16

^a^Data are expressed as post-treatment value less the pretreatment value and are given as mean ± standard deviation (95% confidence interval)

^b^ Obtained by one-way analysis of variance for unadjusted values and by analysis of covariance for adjusted values (for each outcome, the corresponding baseline value and baseline dietary intake were included as covariates)

^c,d^ Different letters demonstrate statistical difference (*P* < 0.05)

All *P* values for pairwise between-group differences were obtained by Bonferroni post-hoc test; only statistically significant results are shown

*UUN* urine urea nitrogen, *TLC* total lymphocyte count

Nitrogen balance (primary outcome) improved and serum creatinine increased in the two ISP groups and they were significant compared with the control group. However, after controlling for potential covariates in ANCOVA models, changes in these indices were statistically significant compared with the control groups (*P* = 0.008, *P* = 0.005 for both nitrogen balance and serum creatinine, respectively). Changes in anthropometric outcomes over the three-week intervention period in ITT populations are also presented in Table 3. At three weeks, there were significant decreases in weight and BMI in each group (all *P* < 0.001). After controlling for potential covariates in ANCOVA models, we found significant differences between groups in terms of three-week changes in all anthropometric outcomes (all *P* ≤ 0.001). Weight loss was observed to be higher in Group C (controls) compared to Group A (ISP + FO). All of these markers are associated with muscle catabolism. However, 24-h UUN, TLC, and 24-h UUC as other muscle catabolism markers were not significant between groups (*P* > 0.05). There was a significant decrease in serum levels of ALT, AST, ALP, TG, cholesterol, and FBS in pairwise comparisons of all three groups but changes of these indices were not statistically significant between groups (*P* > 0.05) (Table 4).

**Table 4:** The effect of treatment on markers of lipid profile, hepatic enzymes, and fasting blood sugar of burn patients during the intervention^a^

Variables	Group A ISP + FO (*n* = 25)	Group B ISP + CO (*n* = 24)	Group C Control (*n* = 24)	*P* value
FBS^b^ (mg/dL)	1.93 ± 1.55^c^ (0.0 to 3.8)	3.15 ± 1.44^c^ (0.85 to 5.44)	3.28 ± 0.71^c^ (2.54 to 4.03)	0.58
TG (mg/dL)	−30.04 ± 64.40^c^ (− 59.36 to − 0.73)	−3.28 ± 84.76^c^ (− 41.86 to 35.29)	1.58 ± 59.84^c^ (− 23.68 to 26.85)	0.28
Total cholesterol (mg/dL)	−44.38 ± 27.93^c^ (− 57.09 to − 31.66)	−26.19 ± 48.44^c^ (− 48.24 to − 4.13)	− 13.75 ± 51.80^c^ (− 35.62 to 8.12)	0.07
ALT^b^ (IU/L)	3.42 ± 1.09^c^ (2.79 to 4.05)	3.27 ± 1.47^c^ (2.45 to 4.08)	3.25 ± 0.83^c^ (2.86 to 3.64)	0.84
AST^b^ (IU/L)	2.49 ± 1.26^c^ (1.51 to 3.46)	3.23 ± 0.84^c^ (2.69 to 3.77)	3.24 ± 1.18^c^ (2.48 to 3.99)	0.16
ALP (IU/L)	57.76 ± 15.88^c^ (24.63 to 90.8)	56.17 ± 25.06^c^ (30.87 to 90.46)	63.70 ± 12.80^c^ (37.22 to 90.19)	0.95
Total protein (g/dL)	+ 0.91 ± 0.22^c^ (0.45 to 1.38)	+ 0.89 ± 0.25^c^ (0.37 to 1.4)	+ 0.74 ± 0.27^c^ (0.18 to 1.3)	0.86
Total bilirubin^b^ (mg/dL)	−2.57 ± 0.70^c^ (− 3.31 to − 1.83)	−2.76 ± 0.52^c^ (− 3.40 to − 2.11)	−2.93 ± 0.55^c^ (− 3.40 to 2.11)	0.10
Direct bilirubin^b^ (mg/dL)	−2.04 ± 0.22^c^ (− 2.14 to − 1.93)	−2.04 ± 0.20^c^ (− 2.14 to − 1.95)	−2.07 ± 0.19^c^ (− 2.15 to − 1.99)	0.75

*FBS* fasting blood sugar, *TG* triglyceride, *ALT* alanine aminotransferase, *AST* aspartate aminotransferase, *ALP* alkaline phosphatase

^a^Data are expressed as post treatment value less the pretreatment value and are given as mean ± standard deviation (95% confidence interval). Values for continuous variables, as mean ± standard deviation if normal distribution or we get a Ln for skewed distribution ^b^

^c,d^ Different letters demonstrate statistical difference (*P* < 0.05)

All *P* values for pairwise between-group differences were obtained by post hoc test; only statistically significant results are shown.

### Effect of treatment on anemia markers

Concentrations of anemia status indexes at the end of treatment phase are shown in Table 5. No significant differences were found in hemoglobin and other CBC markers in pairwise comparisons of all three groups and three-week changes of these indices between groups (*P* > 0.05) although these values tended to be higher in the ISP groups. These data showed that burn frequently results in acute anemia, which was seen in most patients until discharge unless modified by blood transfusion practices. There were no serious adverse events that occurred during the study due to the study starting on day 5. Clinical investigations with this patient population had to be performed for several days after admission since the peak inflammation response occurred by about the fourth day.

**Table 5:** The effect of treatment on markers of cell blood count of burn patients during the intervention^a^

Variables	Group A ISP + FO (*n* = 25)	Group B ISP + CO (*n* = 24)	Group C Control (*n* = 24)	*P* value
RBC (10^6^/μL)	−1.97 ± 0.23^b^ (−2.46 to 1.47)	−2.22 ± 0.21^b^ (− 2.68 to 1.76)	−2.44 ± 0.21^b^ (− 2.88 to 2.00)	0.58
WBC (10^3^/μL)	− 44.38 ± 14.05^b^ (− 8.59 to − 1.39)	−26.19 ± 18.49^b^ (− 7.12 to 0.27)	−13.75 ± 12.21^b^ (− 9.59 to − 0.58)	0.79
Lymphocyte (%)	+ 3.38 ± 3.00^b^ (− 2.88 to 9.65)	−2.90 ± 2.50^b^ (− 2.32 to 8.13)	−0.20 ± 2.01^b^ (− 4.37 to 3.96)	0.53
Hb (g/dL)	− 3.99 ± 0.58^b^ (− 5.20 to − 2.78)	−4.47 ± 0.57^b^ (− 5.68 to − 3.28)	−5.82 ± 0.55^b^ (− 6.96 to − 4.67)	0.06
HCT (%)	−15.46 ± 1.79^b^ (− 19.21 to − 11.70)	−15.30 ± 1.77^b^ (− 19.00 to − 11.60)	−18.50 ± 1.31^b^ (− 21.22 to − 15.79)	0.28
MCV (FL)	+ 2.28 ± 1.09^b^ (0.00 to 4.56)	+ 0.92 ± 0.58^b^ (0.29 to 2.14)	+ 2.24 ± 0.83^b^ (0.51 to 3.97)	0.46
MCH (Pg)	+ 0.80 ± 0.41^b^ (− 0.05 to 1.67)	+ 0.51 ± 0.24^b^ (0.00 to 1.02)	+ 0.71 ± 0.59^b^ (− 0.51 to 1.94)	0.90
MCHC (g/dL)	+ 0.05 ± 0.37^b^ (− 0.72 to 0.84)	−0.20 ± 0.43^b^ (− 1.10 to 0.70)	−0.29 ± 0.21^b^ (− 0.74 to 0.15)	0.75

*RBC* red blood cell count, *WBC* white blood cell count, *HB* hemoglobin, *HCT* hematocrit, *MCV* mean corpuscular volume, *MCH* mean corpuscular hemoglobin, *MCHC* mean corpuscular hemoglobin concentration

^a^Data are expressed as post treatment value less the pretreatment value and are given as mean ± standard deviation (95% confidence interval). Values are presented as means ± SD. All *P* values for pairwise between-group differences were obtained by post hoc test; only statistically significant results are shown

^b^ Different letters demonstrate statistical difference (*p* < 0.05)

## Discussion

To our knowledge, this is the first RCT designed to evaluate the effect of ISP alone or in combination with FO on burn patients. Burn causes malnutrition, poor wound healing, muscle wasting, severe cachexia, frequent infections, and anemia [3–5]. The purpose of nutritional care is maintaining nutritional status to counteract the catabolic status and to promote wound healing through the supply of adequate nutrients. Findings suggest both of the ISP treatments efficiently increased serum creatinine, nitrogen balance, weight, and BMI as markers of nutritional status and muscle catabolism. Our findings showed positive effects of the two ISP groups on nutritional markers, serum creatinine, nitrogen balance (the primary outcomes), weight, and BMI and these were all significantly different from the control group. All of these markers are associated with muscle catabolism. A number of studies have reported on ISP in other clinical conditions. For instance, an improvement in whole-body nitrogen balance and lean body mass has been reported by a large number of investigators [19, 37–40]. However, we believe that more evidence from future well designed RCTs on the benefits of suitable dietary intervention on burn patients is needed to corroborate our findings.

Overall, several mechanisms have been postulated for the potential effects of soy protein on body weight and muscle catabolism. Dietary soy protein has been shown to have the potential to induce hormonally mediated upregulation of muscle protein breakdown [38]. The advantages of soy protein over animal protein intake are controversial in critical illness but would benefit from investigation. It has been shown that ISP is rich in branched chain amino acids such as leucine that can stimulate muscle protein synthesis by rapamycin complex-1, a key signaling protein [41–43]. ISP is also a rich source of arginine, which is a non-essential amino acid that may become conditionally essential during periods of physiological stress [44]. By means of proline, which is hydroxylated to form hydroxyproline, arginine is involved in collagen formation, tissue repair, and wound healing [45]. It is a substrate in the urea cycle and plays a role in nitrogen metabolism, protein, creatinine, and polyamine synthesis [44, 46]. Arginine promotes stimulated T cells and plays a role in cellular immunity and fibroblast proliferation [47]. Branched-chain amino acids and glutamine are other bioactive nutrients that have been seen in the ISP and may decrease urea nitrogen output (appearance) and increase nitrogen balance [48]. Administration of glutamine improves protein balance [49, 50]. Arginine also may increase nitrogen balance [51].

It has been suggested that the isoflavones present in soy protein may lower cholesterol levels in populations consuming high soy diets [52, 53]. A dose-response effect of isoflavones on reducing total cholesterol levels when consumed with a soy protein diet was depicted [54]. In contrast to the present and prior studies on other critical conditions, there was no difference in serum total cholesterol and TG [53, 55–57]. The ISP can upregulate GLUT2, GLUT3, and glucokinase and can induce a reduction in glucose-6-phosphatase expression and lead to a reduction in serum glucose [58, 59]; this might stimulate insulin secretion by activating the cAMP/PKA and PLC/PKC pathways [60]. On the other hand, according to the previous studies, omega-3 content of FO can cause reductions in serum lipids and glucose, which is inconsistent with the results of the current study regarding the decreasing trend in group A [61–63].

Burn causes liver injury and hepatic enzymes can be increased [64]. In this study, the results showed that the ALT, AST, and ALP levels in ISP groups were lower than those of the control group, but there was no significant difference between the groups. To our knowledge, there is no human study for evaluating the effect of ISP on the hepatic enzymes of burn patients and it needs more research. According to the previous studies that have been done on other metabolic conditions, we think that the soy isoflavones, particularly genistein, are responsible for reducing serum liver enzymes [65–68].

Another problem in burn patients that can exacerbate their wellbeing is anemia. Anemia occurs frequently during critical illnesses [5]. The World Health Organization (WHO) defines anemia as a hemoglobin level < 13 and 12 g/dL^-1^ and a hematocrit level < 39% and 36% for adult men and non-pregnant women, respectively [69]. In this study, the markers of CBC were not significantly different between the groups. Ausman et al. showed that low consumption of ISP is correlated with severe anemia in monkeys [70]. Also, omega-3 fatty acids of FO could prevent oxidative damage to specific membrane proteins which could also help to prevent cell lysis [71, 72]. However, this was the first study that evaluated the effect of ISP and FO on burn patients and it needs more studies in anemia after burning to elucidate the final effects on anemia markers.

Soy is a complex of components, including isoflavones, phytate, saponins, conglycinin, and a number of special amino acids, each of which may be responsible for its benefits in this study. The isoflavone and the amino acid constituents seem to be more important in the beneficial effects of ISP. Future studies may elucidate the effects of the other specific components of ISP on wound healing.

The first advantage of our study is that double-blind placebo-controlled RCTs represent the highest level of research evidence. Second, this is the first survey to evaluate the effect of ISP alone or in combination with FO on burn patients. One major limitation of this study was the absence of a treatment group for consuming FO alone. Because of the difficult conditions of sampling in burn patients, low sample size was another drawback of our study that prevented observing significant associations in some cases. On the other hand, this was a single-centered study, so the generalizability of the study findings was limited.

### Trial status

The study was performed from June 2014 through December 2014.

## Conclusions

In summary, the results of this study showed that nutritional support with ISP alone or in combination with FO can decrease muscle catabolism in burn patients. Future research with larger sample sizes and longer durations might be necessary to explore and compare the effect of the individual constituents of ISP and FO on burn injury and to elucidate the exact mechanism of action.

## Abbreviations

ALP: Alkaline phosphatase
ALT: Alanine transaminase
ANCOVA: Analysis of covariance
AST: Aspartate transaminase
BMI: Body mass index
CBC: Cell blood count
CO: Corn oil
FBS: Fasting blood sugar
FO: Flaxseed oil
ISP: Isolated soy protein
ITT: Intention-to-treat analysis
SIRS: Systemic inflammatory response
TG: Triglyceride
TLC: Total lymphocyte count
UUC: 24-h urinary creatinine
UUN: 24-h urinary urea nitrogen excretion
